# lncRNA IGHC*γ*1 Acts as a ceRNA to Regulate Macrophage Inflammation via the miR-6891-3p/TLR4 Axis in Osteoarthritis

**DOI:** 10.1155/2020/9743037

**Published:** 2020-01-17

**Authors:** Pengjun Zhang, Jianmei Sun, Caihong Liang, Bingjie Gu, Yang Xu, Hongying Lu, Bo Cao, Hao Xu

**Affiliations:** ^1^Department of Joint Surgery, The Affiliated Hospital of Qingdao University, Qingdao University, Qingdao 266100, China; ^2^Department of Emergency Surgery, People's Hospital of Rizhao, Rizhao 276800, China; ^3^Department of Chemistry, School of Applied Chemistry, Food and Drug, Weifang Engineering Vocational College, Qingzhou 262500, China; ^4^Department of Cardiovasology, The Affiliated Jiangning Hospital of Nanjing Medical University, Nanjing 210000, China; ^5^Department of Rheumatology and Immunology, Nanjing First Hospital, Nanjing Medical University, Nanjing 210006, China; ^6^National Center for Occupational Safety and Health, NHC, Beijing 100023, China; ^7^Functional Laboratory, Weifang Medical University, Weifang 261000, China; ^8^Department of Emergency Medicine, Affiliated Hospital of Weifang Medical University, Weifang 261000, China

## Abstract

Accumulating data have implicated that long noncoding RNA (lncRNA) plays an important role in osteoarthritis (OA), which may function as a competitive endogenous RNA (ceRNA) of microRNAs (miRNAs). lncRNA IGHC*γ*1 has been demonstrated to regulate inflammation and autoimmunity. Nonetheless, the altering effect of IGHC*γ*1 in OA remains unclear. This study is aimed at investigating the mechanism and function of lncRNA IGHC*γ*1 in OA. CCK-8, EdU, and transwell assays were used to estimate macrophage proliferation and migration. Fluorescence in situ hybridization (FISH) was performed to estimate the local expression of lncRNA IGHC*γ*1 in macrophages. Luciferase reporter assay was adopted to validate the ceRNA role of IGHC*γ*1 as miRNA sponge. lncRNA IGHC*γ*1 was primarily localized in macrophage cytoplasm and upregulated in OA. miR-6891-3p inhibited macrophage proliferation, migration, and inflammatory response by targeting TLR4, while lncRNA IGHC*γ*1 promoted TLR4 expression by functioning as a ceRNA for miR-6891-3p through the NF-*κ*B signal in macrophages. This study strongly supports that lncRNA IGHC*γ*1 regulates inflammatory response via regulating the miR-6891-3p/TLR4/NF-*κ*B axis in macrophages.

## 1. Introduction

Osteoarthritis (OA) is one of the most common degenerative diseases and a major cause of disability in older adults. It leads to a great burden on society and economy. Apart from damages in cartilage and subchondral bone, OA also causes synovitis [1, 2]. The etiology and pathogenesis of OA remain not fully understood up till now. It has been well documented that age, obesity, abnormal anatomical structure, joint trauma history, and excessive use of joints are closely related to OA [3]. Accumulated data have suggested many inflammatory cells and their produced inflammatory mediators contribute to OA pathogenesis, such as IL-6 and TNF-*α* [4, 5]. IL-6 and TNF-*α* can be produced by macrophages, synoviocytes, or articular cartilage itself, which contribute to OA by inducing the expression of metalloproteinases (MMP). In addition, the dysfunction of macrophages has been suggested to play a key role in OA pathogenesis [6]. Therefore, besides tissue engineering, biological therapies by targeting inflammation-associated genes or cells are potential therapeutic strategies for OA patients. Moreover, there are still many challenges for the regeneration of articular cartilage in OA treatment particularly under inflammatory microenvironment.

Emerging studies have shown noncoding RNAs (ncRNAs), for instance, microRNAs (miRNAs), circular RNAs (circRNAs), and long noncoding RNAs (lncRNAs), are involved in OA development and progression [7–9]. A large body of data has demonstrated lncRNA and circRNA can act as competitive endogenous RNAs (ceRNAs) via miRNAs sponge, leading to suppression of miRNAs [10–12]. miRNAs are common ncRNAs involved in regulating autoimmunity and inflammation, which can decrease the expression of targeted mRNAs. Available studies have revealed a variety of miRNAs are aberrantly expressed in OA patients [13, 14]. Our previous study has shown that lncRNA IGHC*γ*1 (also called IGHCgamma1) is involved in regulating arthritis [15], but the altering effect of lncRNA IGHC*γ*1 in OA is unknown. With development in bioinformatics and molecular biology techniques, the ceRNA mechanism based on interactions among lncRNA, miRNA, and mRNA has been extensively elucidated [16, 17]. However, whether lncRNA IGHC*γ*1 functions as a ceRNA in regulating OA needs to be investigated. The current study is aimed at elucidating the role of lncRNA IGHC*γ*1 and its mechanism in OA.

## 2. Materials and Methods

### 2.1. Characteristics of Participants

88 cases and 36 healthy controls adjusted by age and sex are enrolled from the Affiliated Hospital of Qingdao University and People's Hospital of Rizhao, Shandong Province. Patients and controls have all signed the written informed consent. Research is under supervision of the Institutional Ethics Committee of our hospital. Detailed characteristics are presented in Table 1.

### 2.2. Isolation of Peripheral Blood Mononuclear Cells (PBMCs)

We separate PBMCs from cases and controls by Ficoll-Paque density gradient centrifugation. The lymphocyte isolation reagent (Solarbio, Beijing, China) is used for isolation of PBMCs based on the protocol. Cells were harvested for subsequent experiments.

### 2.3. Cell Transfection

THP-1 cell is purchased from ATCC (Porton Down, Salisbury, UK), which is cultured in RPMI 1640 (Invitrogen, NY, USA) with 10% fetal bovine serum and antibiotics. Cells are stimulated by 100 nM PMA for 48 hours and differentiated into macrophage-like cells (called pTHP-1 cells). PcDNA3.1 lentivirus vectors with upregulation or downregulation of lncRNA IGHC*γ*1 and TLR4 are constructed and used to transfect pTHP-1 cells. We purchase miR-6891-3p mimics, inhibitors, and controls from RiboBio (Guangzhou, China). Lipofectamine 2000 (Invitrogen, NY, USA) is adopted for transfection based on the protocol.

### 2.4. Real-Time Polymerase Chain Reaction (PCR)

To extract cytoplasm and nuclear RNAs separately, we adopted the kit for nuclear and cytoplasmic RNA purification (Norgen Biotek, Thorold, Canada). Then, RNAs were quantified by real-time PCR. TRIzol reagent (Invitrogen, CA, USA) was applied. 500 ng RNAs are used for cDNA synthesis, which are isolated from cell lines or PBMCs according to the protocol. mRNAs of IL-6 and TNF-*α*, TLR4, and lncRNA IGHC*γ*1 are assayed by PCR and normalized to GAPDH based on the reaction system. Primers for human genes are shown as follows: TNF-*α*: forward: ATGTGGCAAGAGATGGGGAA, reverse: CTCACACCCCACATCTGTCT; IL-6: forward: AGTCCTGATCCAGTTCCTGC, reverse: CTACATTTGCCGAAGAGCCC; TLR4: forward: CCAGCCTCCTCAGAAACAGA, reverse: TCCCTCCAGCAGTGAAGAAG; IGHC*γ*1: forward: GTGACGGTGTCGTGGAACTC, reverse: GTGTTGCTGGGCTTGTGATT; and GAPDH: forward: AAGGAAATGAATGGGCAGCC, reverse: TAGGAAAAGCATCACCCGGA.

### 2.5. Cell Proliferation Assay

Cell counting kit-8 (CCK-8) (Vazyme Biotech, Nanjing, China) is applied to evaluate the growth of cells. In brief, 2 × 10^3^ cells are seeded into cell cultural plate and activated by LPS (1 *μ*g/ml) for 12, 24, and 48 hrs. The absorption is determined following the protocol of CCK-8. CellLight 5-ethynyl-2′-deoxyuridine (EdU) is also carried out by use of Apollo567 kit (RiboBio, Guangzhou, China) for cell proliferation determination after stimulation by LPS for 24 hrs.

### 2.6. Enzyme-Linked Immunosorbent Assay (ELISA)

In brief, lncRNA IGHC*γ*1 or TLR4 overexpressed cells (1 × 10^5^/ml) are seeded into 96-well cell culture plate in serum-free medium overnight and then treated by miR-6891-3p mimics or not for 24 hrs. The supernatant was acquired for cytokine determination by use of the ELISA kits. IL-6 and TNF-*α* ELISA kits (R&D, Minnesota, USA) are adopted based on the protocol as previously described [18].

### 2.7. Western Blot

30 *μ*g/channel proteins are used for analysis. We extract proteins from pTHP-1 cells, which are lysed with RIPA buffer (Beyotime Technology, Shanghai, China). Proteins are separated by gel electrophoresis and recognized by TLR4, phosphorylated NF-*κ*B (p-NF-*κ*B) (Santa Cruz Biotechnology, CA, USA), GAPDH (CST, Boston, USA), and *β*-actin antibodies (Abcam, Cambridge, UK).

### 2.8. Fluorescence In Situ Hybridization (FISH)

We carry out FISH assay to estimate the local expression of lncRNA IGHC*γ*1 in pTHP-1 cells. 5 × 10^5^/ml cells are fixed with paraformaldehyde (4%). After protease-K incubation, pTHP-1 cells are dehydrated in a gradient manner with diverse concentrations of ethanol and then hybridized by IGHC*γ*1 probe labeled by fluorescence. DAPI (Life Technologies, Carlsbad, USA) is used to stain the nucleus. The fluorescence is scanned by a microscope.

### 2.9. Luciferase Reporter Assay

293T cells (5 × 10^5^/ml) are cultured overnight and transfected with miR-6891-3p inhibitors or mimics or controls and/or lv-TLR4-WT or lv-TLR4-MT or lv-IGHC*γ*1-WT or lv-IGHC*γ*1-MT reporter plasmids (150 ng) with lipofectamine 2000. pTHP-1 cells are lysed after transfection, and luciferase activity is determined by the Picagene Dual SeaPansy luminescence kit (Toyo Ink, Japan).

### 2.10. NF-*κ*B Activity Assay

The activity of transcriptional factor NF-*κ*B is detected in a whole cell extract based on the protocol of the ELISA-based kit (Active Motif, CA, USA). Briefly, lncRNA IGHC*γ*1 overexpressed and the control plasmids were used to transfect macrophages. After that, 1 × 10^5^/ml macrophages were cultured in serum-free medium and administrated into miR-6891-3p mimics and the control vectors for 24 hours. Lastly, cells were harvested and used for the detection of NF-*κ*B activity according to the kit instructions mentioned above. Individual experiments were repeated for at least three times.

### 2.11. RNA Binding Protein Immunoprecipitation (RIP) Assay

The Magna RIP Kit (Millipore, Bedford, USA) is used for analysis. Cell lysates were immunoprecipitated using Ago2 antibody or IgG in the buffer. The expression of IGHC*γ*1 in immunoprecipitates is detected by real-time PCR.

### 2.12. Statistical Analysis

Data are presented as mean ± SE. ANOVA or unpaired Student's *t*-test is adopted for comparisons. GraphPad Prism (San Diego, CA, USA) and SPSS software (Chicago, IL, USA) are applied in this study.

## 3. Results

### 3.1. lncRNA IGHC*γ*1 Was Elevated in OA and Macrophage Cell Lines

**Figure 1:**
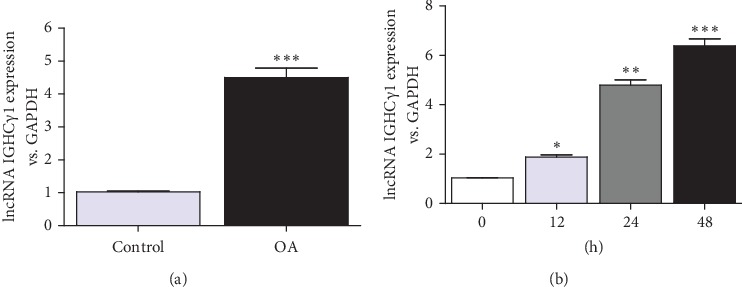
lncRNA IGHC*γ*1 was upregulated in OA. (a) Increased expression of lncRNA IGHC*γ*1 relative to GAPDH in PBMCs from OA (^∗∗∗^*P* < 0.001; frequencies of cases and controls: 88/36). (b) LPS (1 *μ*g/ml) activated macrophages and promoted lncRNA IGHC*γ*1 expression in cells (*N* = 3; ^∗^*P* < 0.05; ^∗∗^*P* < 0.01; ^∗∗∗^*P* < 0.001).

lncRNA IGHC*γ*1 was found to be highly expressed in PBMCs of OA patients (Figure 1(a)). Besides, it was demonstrated that the expression of lncRNA IGHC*γ*1 was upregulated in a time-dependent manner in PMA-induced THP-1 (pTHP-1) macrophages, which were activated by LPS for 12, 24, and 48 hours (Figure 1(b)). It was well known that macrophages are major immune cells involved in OA. Accordingly, we hypothesized that lncRNA IGHC*γ*1 might affect macrophage-mediated autoimmunity and inflammation regulation in OA.

### 3.2. lncRNA IGHC*γ*1 Promoted the Proliferation of Macrophages

**Figure 2:**
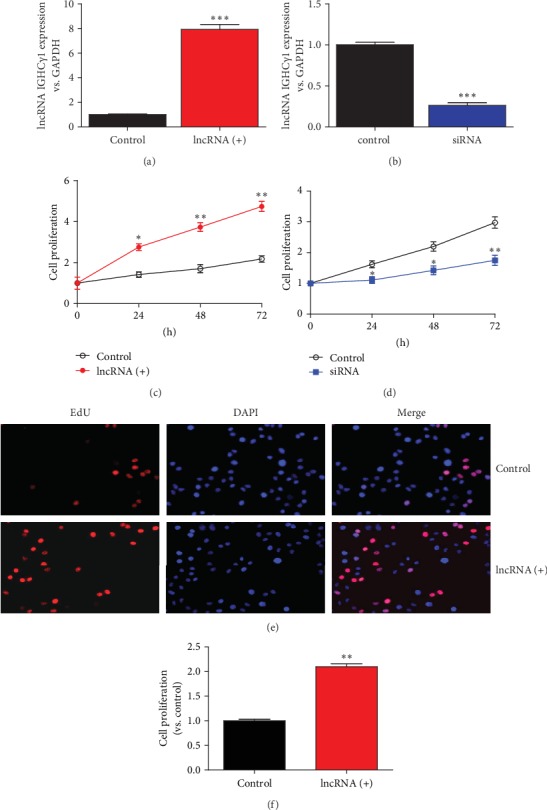
lncRNA IGHC*γ*1 promoted macrophage proliferation and migration. (a) Real-time PCR showed lncRNA IGHC*γ*1 expression after overexpression in macrophages (*N* = 3; ^∗∗∗^*P* < 0.001). (b) Real-time PCR presented IGHC*γ*1 expression after knockdown in macrophages (*N* = 3; ^∗∗∗^*P* < 0.001). (c) CCK-8 revealed that lncRNA IGHC*γ*1 enhanced the proliferation of macrophages (*N* = 3; ^∗^*P* < 0.05; ^∗∗^*P* < 0.01). (d) siRNA of IGHC*γ*1 inhibited macrophage proliferation assayed by CCK-8 (*N* = 3; compared with the control group, ^∗^*P* < 0.05; ^∗∗^*P* < 0.01). (e) IGHC*γ*1 promoted macrophage proliferation when it was upregulated in cells assayed by EdU (representative pictures of EdU assays). (f) lncRNA IGHC*γ*1 promoted macrophage proliferation (data of three repeated EdU experiments; compared with controls, ^∗∗^*P* < 0.01).

Figures 2(a) and 2(b) showed lncRNA IGHC*γ*1 expression in macrophages. The proliferation of macrophages was enhanced when lncRNA IGHC*γ*1 was upregulated in cells, whereas cell proliferation was inhibited when lncRNA IGHC*γ*1 was knocked down in macrophages demonstrated by CCK-8 and EdU assay (Figures 2(c)–2(f)). Taken together, lncRNA IGHC*γ*1 could promote the growth of macrophages *in vitro*.

### 3.3. lncRNA IGHC*γ*1 Functioned as ceRNA for miR-6891-3p in Macrophages

**Figure 3:**
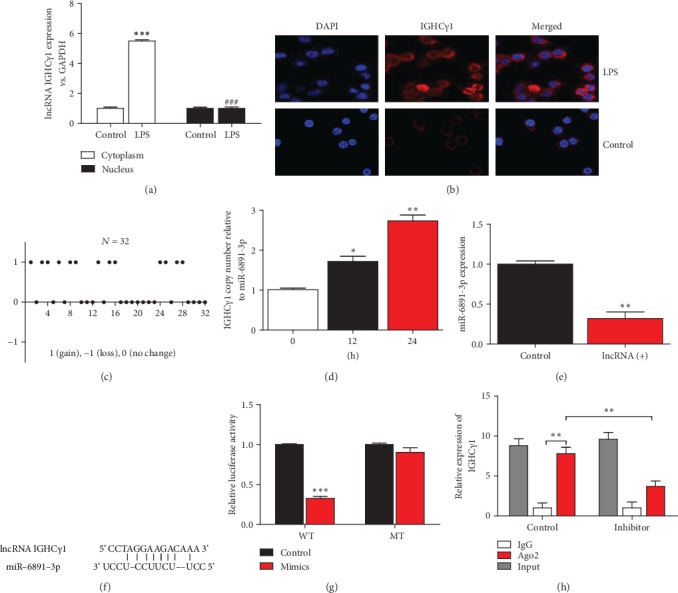
lncRNA IGHC*γ*1 functioned as ceRNA via binding miR-6891-3p. (a) Real-time PCR revealed lncRNA IGHC*γ*1 primarily existed in the cytoplasm of macrophages (these data represented 3 independent experiments; compared with controls, ^∗∗∗^*P* < 0.001; compared with the LPS-treated macrophage group, ^###^*P* < 0.001). (b) FISH also showed lncRNA IGHC*γ*1 was mainly expressed in pTHP-1 cytoplasm (pictures represent one of three repeated FISH assays). (c) Increased copy number gains of lncRNA IGHC*γ*1 in OA PBMC samples (*N* = 32). (d) Increased copy number gains of lncRNA IGHC*γ*1 relative to miR-6891-3p in pTHP-1 cells stimulated by LPS (*N* = 3; ^∗^*P* < 0.05; ^∗∗^*P* < 0.01). (e) As shown by real-time PCR, the expression of miR-6891-3p in macrophages was significantly reduced when lncRNA IGHC*γ*1 was overexpressed (*N* = 3; ^∗∗^*P* < 0.01). (f) The seed sequence of miR-6891-3p recognized by lncRNA IGHC*γ*1 (data were screened in database of starBase). (g) Decreased luciferase activity in lncRNA IGHC*γ*1 WT transfected cells but not lncRNA IGHC*γ*1 MT cells (*N* = 3; ^∗∗∗^*P* < 0.001). (h) RIP assay showed IGHC*γ*1 in immunoprecipitates. Cell lysates were immunoprecipitated by use of Ago2 antibody and IgG. IGHC*γ*1 expression is determined by real-time PCR (*N* = 3; ^∗∗^*P* < 0.01).

lncRNA IGHC*γ*1 was mainly expressed in pTHP-1 cytoplasm demonstrated by real-time PCR and FISH assay (Figures 3(a) and 3(b)), which suggested lncRNA IGHC*γ*1 might confer its effect by combining with other RNAs or proteins in the cytoplasm of macrophages. High lncRNA IGHC*γ*1 expression in OA PBMCs was partly due to increased copy number gains as shown in Figure 3(c). LPS could also increase the copy number of lncRNA IGHC*γ*1 relative to miR-6891-3p in pTHP-1 macrophages (Figure 3(d)). Thirdly, lncRNA IGHC*γ*1 was demonstrated to be negatively related to miR-6891-3p regarding their expression in macrophages determined by real-time PCR (Figure 3(e)). In order to further investigate whether the two noncoding RNAs can interact with each other by base complementary binding, we performed bioinformatics analysis. lncRNA IGHC*γ*1 was predicted to interact with miR-6891-3p after screening in database of starBase (http://starbase.sysu.edu.cn/). It could specifically recognize the seed sequence of miR-6891-3p (Figure 3(f)). We hypothesized lncRNA IGHC*γ*1 could act as a ceRNA of miR-6891-3p in macrophages. The dual-luciferase reporter assay revealed that lncRNA IGHC*γ*1 could target miR-6891-3p (Figure 3(g)). Moreover, RIP assay showed IGHC*γ*1 could be detected in the immunoprecipitates as demonstrated by real-time PCR. Taken together, lncRNA IGHC*γ*1 could function as a ceRNA of miR-6891-3p in macrophages.

### 3.4. Downregulation of miR-6891-3p Enhanced Cell Proliferation and Migration of Macrophages

**Figure 4:**
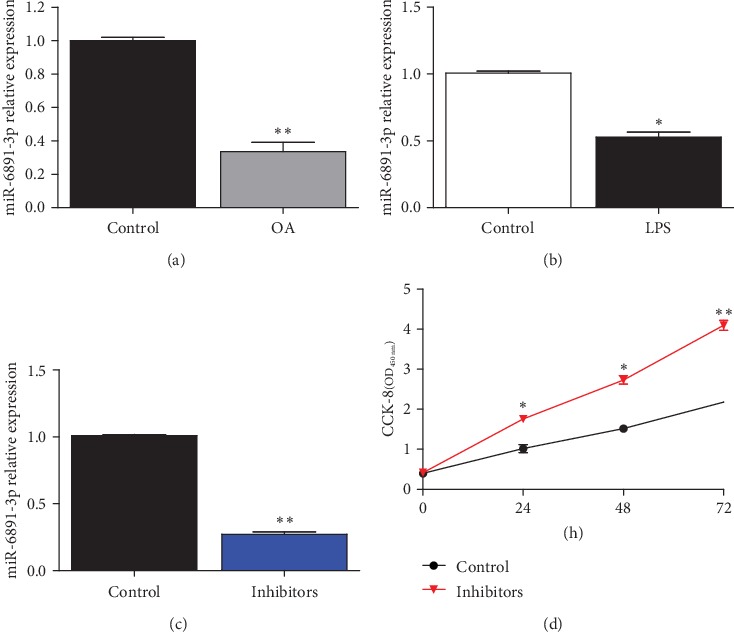
miR-6891-3p promoted macrophage proliferation. (a) miR-6891-3p was decreased in PBMCs of OA patients (cases/controls: 36/30; ^∗∗^*P* < 0.01). (b) Reduced miR-6891-3p in macrophages activated by LPS (1 *μ*g/ml) (*N* = 3; ^∗^*P* < 0.05). (c) As demonstrated by real-time PCR, miR-6891-3p inhibitors efficiently inhibited its expression in macrophages (*N* = 3; ^∗∗^*P* < 0.01). (d) CCK-8 showed elevated proliferation of pTHP-1 cells administrated with miR-6891-3p inhibitors (*N* = 3; ^∗^*P* < 0.05; ^∗∗^*P* < 0.01).

miR-6891-3p has been reported to be a potential regulator in inflammation and immunity [19]. Significantly reduced miR-6891-3p was also demonstrated in OA PBMCs and pTHP-1 cells under stimulation of LPS (Figures 4(a) and 4(b)). To elucidate its functions in OA, we evaluated the influence of miR-6891-3p on macrophage proliferation and migration by use of inhibitors of miR-6891-3p. The real-time PCR showed inhibitors of miR-6891-3p could efficiently inhibit miR-6891-3p expression in macrophages (Figure 4(c)). After downregulation of miR-6891-3p, pTHP-1 cell proliferation was significantly promoted as demonstrated by CCK-8 analysis (Figure 4(d)). Taken together, downregulation of miR-6891-3p promoted macrophage proliferation *in vitro*.

### 3.5. TLR4 Was a Target of miR-6891-3p

**Figure 5:**
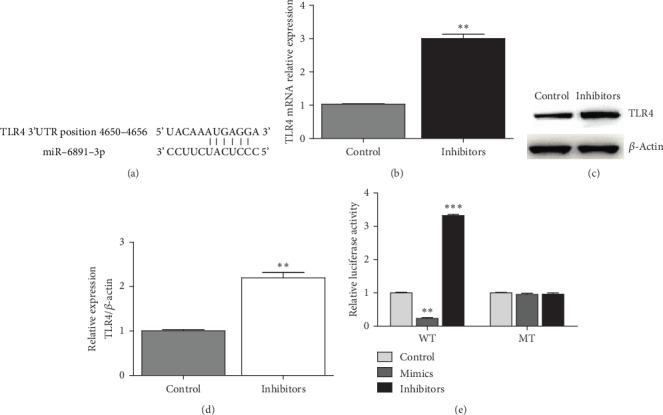
miR-6891-3p regulated in a targeted manner TLR4 in macrophages. (a) The binding sequence of TLR4 3′UTR recognized by miR-6891-3p (information acquired in TargetScan database). (b) miR-6891-3p inhibitors promoted the mRNA expression of TLR4 demonstrated by real-time PCR (*N* = 3; ^∗∗^*P* < 0.01). (c) miR-6891-3p inhibitors promoted TLR4 expression demonstrated by western blot (representative figure for western blot). (d) TLR4 protein expression demonstrated by densitometry of the western blot (*N* = 3; ^∗∗^*P* < 0.01). (e) The luciferase reporter assay demonstrated the luciferase activity of WT TLR4 3′UTR was significantly restrained by miR-6891-3p mimics but not miR-6891-3p inhibitors (*N* = 3; ^∗∗^*P* < 0.01; ^∗∗∗^*P* < 0.001).

Here, TLR4 was predicted to be the potential targeted gene of miR-6891-3p scanned in TargetScan database (http://www.targetscan.org). The 3′UTR of TLR4 contains binding sequence of miR-6891-3p (Figure 5(a)). Downregulation of miR-6891-3p increased the expression of TLR4 in macrophages, which had been demonstrated by PCR and western blot determination (Figures 5(b)–5(d)). Moreover, the luciferase activity of wild-type (WT) TLR4 3′UTR was significantly inhibited by miR-6891-3p mimics but significantly enhanced by miR-6891-3p inhibitors (Figure 5(e)). Nevertheless, miR-6891-3p did not affect the luciferase activity of mutant TLR4 3′UTR. Taken together, TLR4 was a target of miR-6891-3p.

### 3.6. lncRNA IGHC*γ*1 Promoted Inflammation by Regulating miR-6891-3p/TLR4 in Macrophages

**Figure 6:**
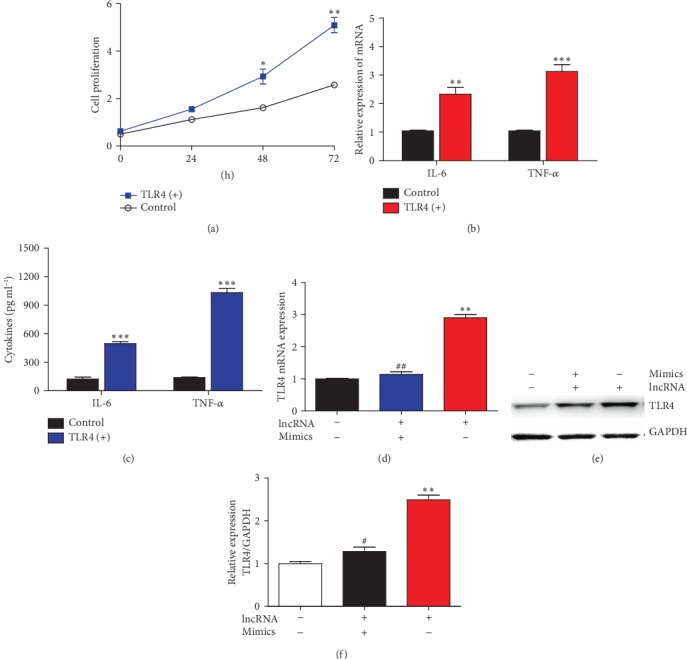
lncRNA IGHC*γ*1 regulated TLR4 expression as a ceRNA of miR-6891-3p in macrophages. (a) Upregulation of TLR4 significantly enhanced the proliferation of macrophages at 48 h and 72 h demonstrated by CCK-8 (*N* = 3; ^∗^*P* < 0.05; ^∗∗^*P* < 0.01). (b) Overexpression of TLR4 promoted IL-6 and TNF-*α* mRNAs in macrophages (*N* = 3; ^∗∗^*P* < 0.01; ^∗∗∗^*P* < 0.001). (c) Overexpression of TLR4 promoted IL-6 and TNF-*α* production in the supernatant of macrophages (*N* = 3; ^∗∗∗^*P* < 0.001). (d) The upregulation of TLR4 mRNA by lncRNA IGHC*γ*1 was significantly suppressed by miR-6891-3p mimics as demonstrated by real-time PCR (*N* = 3; compared with the -/- group, ^∗∗^*P* < 0.01; in contrast to the +/- group, ^##^*P* < 0.01). (e) The upregulation of TLR4 protein by lncRNA IGHC*γ*1 was significantly inhibited by miR-6891-3p mimics as demonstrated by western blot (representative picture for western blot). (f) The densitometry of TLR4 protein detected by western blot (*N* = 3; compared with the -/- group, ^∗∗^*P* < 0.01; compared with the +/- group, ^#^*P* < 0.05).

The CCK-8 assay showed that upregulation of TLR4 enhanced the proliferation of macrophages (Figure 6(a)). Besides, overexpression of TLR4 could promote IL-6 and TNF-*α* expression in macrophages (Figures 6(b) and 6(c)), which suggested TLR4 played a crucial role in macrophage inflammatory response. Subsequently, we performed rescue tests using miR-6891-3p mimics to rescue the intermediate effect of miR-6891-3p in the lncRNA IGHC*γ*1-miR-6891-3p-TLR4 axis. The upregulation of TLR4 by lncRNA IGHC*γ*1 could be suppressed with miR-6891-3p mimics as demonstrated by real-time PCR and western blot (Figures 6(d)–6(f)). Taken together, lncRNA IGHC*γ*1 aggravated TLR4-mediated inflammation by acting as a ceRNA for miR-6891-3p in pTHP-1 macrophages.

### 3.7. lncRNA IGHC*γ*1 Acted as a ceRNA for miR-6891-3p via NF-*κ*B

**Figure 7:**
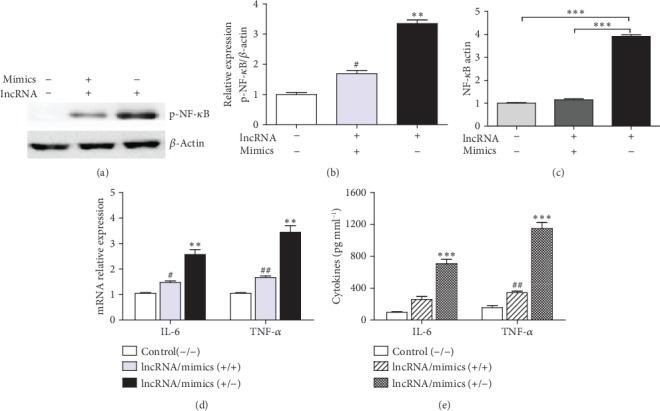
lncRNA IGHC*γ*1 functioned as a ceRNA for miR-6891-3p through NF-*κ*B signaling. (a) lncRNA IGHC*γ*1 promoted NF-*κ*B phosphorylation by sponging miR-6891-3p, while miR-6891-3p mimics rescued its inhibitory effect on NF-*κ*B phosphorylation in macrophages (representative western blot assay). (b) The densitometry of p-NF-*κ*B determined by western blot (*N* = 3; in contrast to the -/- group, ^∗∗^*P* < 0.01; compared with the +/- group, ^#^*P* < 0.05). (c) lncRNA IGHC*γ*1 increased NF-*κ*B activity by acting as ceRNA for miR-6891-3p, whereas miR-6891-3p mimics rescued its effect on NF-*κ*B in pTHP-1 cells (*N* = 3; ^∗∗∗^*P* < 0.001). (d) Expression of IL-6 and TNF-*α* mRNAs in macrophages (*N* = 3; compared with the control (-/-) group, ^∗∗^*P* < 0.01; in contrast to the lncRNA/mimics (+/-) group, ^#^*P* < 0.05; ^##^*P* < 0.01). (e) IL-6 and TNF-*α* in the supernatant of macrophages (*N* = 3; in contrast to the control (-/-) group, ^∗∗∗^*P* < 0.001; compared with the lncRNA/mimics (+/-) group, ^##^*P* < 0.01).

NF-*κ*B is a downstream signaling molecule and a key transcriptional factor involved in regulating TLR4-mediated autoimmune and inflammation. Therefore, the effect of lncRNA IGHC*γ*1 on the downstream signaling pathway of TLR4 was investigated. lncRNA IGHC*γ*1 enhanced NF-*κ*B phosphorylation by sponging miR-6891-3p, while miR-6891-3p mimics could inhibit NF-*κ*B activation by rescuing the proinflammatory effect of lncRNA IGHC*γ*1 in macrophages (Figures 7(a) and 7(b)). In addition, the activity of NF-*κ*B in macrophages was estimated. As shown in Figure 7(c), NF-*κ*B activity was significantly enhanced in pTHP-1 cells with lncRNA IGHC*γ*1 upregulation, whereas miR-6891-3p mimics could reduce the activation of NF-*κ*B. Moreover, lncRNA IGHC*γ*1 promoted the generation of IL-6 and TNF-*α* cytokines in the downstream of the NF-*κ*B pathway in macrophages (Figures 7(d) and 7(e)). As a result, it could be concluded that lncRNA IGHC*γ*1 acted as a ceRNA for miR-6891-3p and thus induced NF-*κ*B activation and downstream cytokine production in macrophages.

## 4. Discussion

lncRNAs belong to noncoding RNAs, which do not encode proteins but possess important biological activity. Accumulating data have suggested lncRNAs are crucial noncoding RNAs involved in regulating cancer, autoimmunity, and inflammation, such as lincRNA-Cox2 and lncRNA-Dreh [20–22]. During the past few years, lncRNA has been demonstrated to be involved in OA with abnormal expression and/or dysregulated functions, particularly in inflammatory cells such as macrophages [23, 24]. Those differentially expressed lncRNAs are specific in OA and may be used as diagnostic or therapeutic targets in the future. lncRNA IGHC*γ*1 is an aberrantly expressed lncRNA in inflammatory arthritis [15]. However, the precise role of lncRNA IGHC*γ*1 in OA is not fully elucidated. In the current study, lncRNA IGHC*γ*1 has been found to be upregulated in OA. It enhances the proliferation of macrophages *in vitro*. Most interestingly, lncRNA IGHC*γ*1 is capable of acting as a ceRNA for miR-6891-3p in macrophages. Furthermore, lncRNA IGHC*γ*1 aggravates inflammation via regulating the miR-6891-3p/TLR4/NF-*κ*B axis in macrophages. These findings are useful for investigating biological markers for OA treatment.

As a kind of noncoding RNA, lncRNA can function as ceRNA and restrain miRNA by lncRNA-miRNA sponge in cancer, cardiovascular disease, and so on [25, 26]. Several functional lncRNAs have been demonstrated to influence osteoblast differentiation and OA pathogenesis through lncRNA-miRNA-mRNA ceRNA mechanism [27–30]. Some lncRNAs can affect the degradation of the extracellular matrix of chondrocytes by functioning as ceRNAs of specific miRNAs and thus participate in the development and progression of OA, such as lncRNAs of HOTTIP, MEG3, and XIST [28, 29, 31]. Some lncRNAs have been well documented to regulate the differentiation of osteoblasts via the lncRNA-miRNA ceRNA network [27]. Besides, Fan et al. have found that DANCR can regulate the progression of OA via targeting miR-577 through ceRNA mechanism [32]. Moreover, the study by Mao et al. shows the evidence that lncRNA HOTAIR is involved in OA pathogenesis by regulating synovial inflammation and proliferation and apoptosis of synoviocytes [33]. However, whether lncRNAs regulate inflammation in OA based on the lncRNA-miRNA ceRNA network warrants to be investigated. In this study, lncRNA IGHC*γ*1 has been firstly revealed to be dysregulated in OA, which is capable of acting as a ceRNA for miR-6891-3p and promoting inflammation in macrophages. As a result, all findings strongly support that the lncRNA-miRNA network plays a critical role in OA.

Accumulating evidence has suggested miRNAs exert significant effects on the development of OA by regulating targeted genes at the posttranscriptional level. miRNAs are involved in articular cartilage damage and repair, extracellular matrix degradation, arthritis, and maintenance of bone homeostasis [34–36]. Some established miRNAs dysregulated in OA have been documented to regulate in a targeted manner certain toll-like receptors (TLRs), the well-known pattern recognition receptors (PRRs) participating in innate immunity [37–39]. miR-6891-3p has been implicated to regulate in a targeted manner TLRs in inflammatory arthritis [19]. Here, we hypothesize miR-6891-3p is involved in OA pathogenesis by targeting TLR4, because TLR4 is predicted to be the targeted gene of miR-6891-3p suggested by bioinformatics analysis. We have demonstrated that miR-6891-3p is dysregulated in OA and macrophages, and lncRNA IGHC*γ*1 can function as a miR-6891-3p sponge and influence the effect of TLR4 on macrophage inflammation *in vitro*.

It has been well documented that macrophages play a critical role in inflammatory response and autoimmunity against virus infection and tumorigenesis, which are also major cells involved in OA pathogenesis [37]. TLRs are critical PRRs mainly expressed on the membrane or in macrophages. A number of studies have implicated that TLR-mediated biological effects in macrophages can maintain or expand inflammatory Th1 and Th17 cell response, such as TLR2, TLR3, TLR4, and TLR9 [40, 41]. Moreover, TLR-mediated inflammation and immune regulation in macrophages are involved in OA development and progression, and TLR4 is one of the most important ones [42–44]. TLR4-mediated inflammatory response in macrophages induces high inflammatory state characteristic of increased inflammatory cytokines in bone microenvironment, such as IL-6 and TNF-*α*. Those inflammatory cytokines can lead to bone inflammation and bone injuries in OA. We have further elucidated the critical role of TLR4 in macrophage inflammatory response in this study. It shows evidence that TLR4 is a target of miR-6891-3p, while lncRNA IGHC*γ*1 can regulate TLR4 via lncRNA-miRNA sponge in macrophages. We have demonstrated a lncRNA IGHC*γ*1-miR-6891-3p-TLR4 axis in OA pathogenesis, which serves as target for the treatment of OA patients.

Although lncRNA IGHC*γ*1 has been documented to aggravate TLR4-mediated inflammation in macrophages via sponging miR-6891-3p *in vitro*, the effect of lncRNA IGHC*γ*1 as a ceRNA warrants being confirmed in *in vivo* studies including animal models of OA. In addition, although an IGHC*γ*1/miR-6891-3p/TLR4/NF-*κ*B axis has been demonstrated in OA pathogenesis, more studies are needed to explore the downstream signaling points of the IGHC*γ*1/miR-6891-3p/TLR4 axis in regulating macrophage inflammation.

In summary, findings in this study will help to understand OA pathogenesis. The lncRNA IGHC*γ*1/miR-6891-3p/TLR4/NF-*κ*B axis will offer potential biomarkers for OA treatment. Nevertheless, more studies are needed to fully elucidate the molecular mechanism of lncRNA IGHC*γ*1 in OA, especially studies *in vivo*.

## Figures and Tables

**Table 1:** Characteristics of participants.

	Case	Control
Number	88	36
Age (years)	52.2 ± 15.8	50.3 ± 13.1
Gender (F/M)	50/38	20/16
CRP (mg/l)	23.8 ± 4.1	5.8 ± 1.7
ESR (mm/h)	28.4 ± 5.7	14.1 ± 5.9

## Data Availability

Data in this study is available from the corresponding authors upon request.
